# *Bacillus cereus* MH778713 elicits tomato plant protection against *Fusarium oxysporum*

**DOI:** 10.1111/jam.15179

**Published:** 2021-07-06

**Authors:** Verónica Ramírez, Javier Martínez, María del Rocio Bustillos‐Cristales, Dolores Catañeda‐Antonio, José‐Antonio Munive, Antonino Baez

**Affiliations:** ^1^ Centro de Investigaciones en Ciencias Microbiológicas Instituto de Ciencias Benemérita Universidad Autónoma de Puebla Puebla México; ^2^ Facultad de Ciencias Químicas Benemérita Universidad Autónoma de Puebla Puebla México; ^3^ Centro de Investigación en Dispositivos Semiconductores Instituto de Ciencias Benemérita Universidad Autónoma de Puebla Puebla México

**Keywords:** agriculture, Bacillus, biocontrol, fungi, plant diseases

## Abstract

**Aim:**

The genus *Fusarium* comprises plant pathogenic species with agricultural relevance. *Fusarium* *oxysporum* causes tomato wilt disease with significant production losses. The use of agrochemicals to control the *Fusarium* wilt of tomato is not environmentally friendly. *Bacillus* species, as biocontrol agents, provide a safe and sustainable means to control *Fusarium*‐induced plant diseases. In this study, the ability of *Bacillus* *cereus* MH778713, a strain isolated from root nodules of *Prosopis laevigata*, to protect tomato plants against *Fusarium* wilt was evaluated.

**Methods and results:**

*Bacillus* *cereus* MH778713 and its volatiles inhibited the radial growth of *F*. *oxysporum* and stimulated tomato seedling growth in *in vitro* and *in vivo* tests. When tomato plants growing in the greenhouse were inoculated with *B*. *cereus* MH778713, the percentage of wilted plants decreased from 96% to 12%, indicating an effective crop protection against *Fusarium* wilt. Among the metabolites produced by *B*. *cereus* MH778713, hentriacontane and 2,4‐di‐tert‐butylphenol promoted tomato seedling growth and showed antifungal activity against the target pathogen.

**Conclusion:**

The inoculation of *B*. *cereus* MH778713 on tomato seedlings helped plants to manage *Fusarium* wilt, suggesting the potential of *B*. *cereus* MH778713 as a biocontrol agent.

**Significance and Impact of the Study:**

These results complement our previous studies on chromium tolerance and bioremediation traits of *B*. *cereus* MH778713 by highlighting the potential of this metal‐resistant micro‐organism to boost crop growth and disease resistance.

## INTRODUCTION

*Bacillus* species exhibit an arsenal of physiological capabilities that allow them to live in numerous natural environments. Several species of *Bacillus* have been identified as plant‐growth‐promoting bacteria (PGPB) and/or biocontrol agents. They can stimulate plant growth and confer plant tolerance to biotic and abiotic stresses (Lee et al., 2012; Park et al., 2013; Zhang et al., 2008). PGPB stimulate the growth of plants by producing phytohormones, acquiring nutrients such as phosphorous and nitrogen, and controlling pathogens through the production of antimicrobial compounds and lipopeptides (Khan et al., 2018; Ryu et al., 2003). When some root endophytic *Bacillus* are applied to seeds or seedlings, a state of induced systemic resistance (ISR) is triggered in the plant (Park et al., 2013; Ryu et al., 2004). This ISR response protects plants from different stresses such as infection by pathogens, drought and salt stress. Thus, *Bacillus cereus* AR156, *Bacillus subtilis* GB03 and *Bacillus amyloliquefaciens* IN937a have been used to reduce disease severity of fruit/plants. These *Bacillus* strains are common constituents in commercially available products. The inoculation of these *Bacillus* strains on fruit/plants has been shown to initiate an ISR response mediated by the production of volatile and nonvolatile organic compounds (Ryu et al., 2004; Wang et al., 2013). *Bacillus* *subtilis* G8, *B*. *subtilis* C9 *and B*. *amyloquefaciens* have been previously shown to inhibit pathogenic fungi like *Rhizoctonia solani*, *Sclerotinia sclerotiorum* and *Botrytis cinerea* by the production of volatile organic compounds (Gao et al., 2018; Gotor‐Vila et al., 2017; Islam et al., 2012).

*Bacillus cereus* MH778713 isolated from *Prosopis laevigata* nodules helps *P*. *laevigata* plants to tolerate chromium toxicity and promotes plant‐growth in nitrogen‐free Jensen's medium (Ramírez et al., 2019). When Cr‐stressed seed of *Arabidopsis thaliana* and *P*. *laevigata* are exposed to the blend of volatiles produced by *B*. *cereus* MH778713, seed dormancy is reversed. Among the metabolites released by *B*. *cereus* MH778713, heneicosane, 2,4‐di‐tert‐butylphenol, hentriacontane and tetracosane were found to be abundant and able to induce seed dormancy‐breaking (Ramírez et al., 2020). Furthermore, those compounds were able to stimulate the growth of *P*. *laevigata* seedlings by increasing root and shoot length. *Bacillus* *cereus* MH778713 has also been effective in stimulating plant‐growth of legumes and protecting them from Cr‐stress, however, the ability to stimulate and protect nonlegume crops has not previously been studied. Therefore, tomato plants were selected in this work to explore the ability of *B*. *cereus* MH778713 as a biological control agent.

Among the causal agents of infectious diseases of crop plants, phytopathogenic fungi play a dominant role by causing devastating crop yield losses. Some members of the genus *Fusarium* are phytopathogens, widely distributed in soil, with agricultural relevance, causing economic losses (Khan et al., 2017). The fungus *Fusarium oxysporum* f. sp. *lycopersici* causes the tomato wilt disease with significant production losses in Mexico (Isaac et al., 2018; Sanchez‐Peña et al., 2010). Excessive use of fungicides (prochloraz, carbendazim, thiabendazole and mancozeb) to control the *Fusarium* wilt disease, can cause resistance in the pathogen and might be not friendly with the environment (Liu et al., 2019; Runkle et al., 2017; Vinggaard et al., 2006). Some agrochemical residues on food represent a risk to human health (Jallow et al., 2017; Nascimento et al., 2017). New alternatives to control fungal diseases include the use of antagonistic micro‐organisms capable of produce antifungal compounds and hydrolysing enzymes. Microbial antagonist strains can release nonvolatile and volatile organic compounds, some of which exhibit inhibitory activity against plant pathogens (Khan et al., 2018; Romeiro et al., 2010). *Bacillus*, *Pseudomonas* and *Streptomyces* genus play an important role in the biological control of fungal pathogens. Nonpathogenic soil *Bacillus* species offer several advantages over other organisms as they form endospores and hence can tolerate extreme pH, temperature and osmotic conditions (Ashwini & Srividya, 2014). Thus, biological control of tomato wilt using *Bacillus* species could provide an effective, safe and sustainable means to control *F*. *oxysporum*‐induced plant diseases (Miljaković et al., 2020). In this study, the ability of *B*. *cereus* MH778713 to protect tomato plants against *F*. *oxysporum* wilt was tested. *B*. *cereus* MH778713 and its volatiles inhibited the radial growth of *Fusarium* and stimulated plant growth *in vitro* tests. A beneficial plant–microbe interaction was observed since *B*. *cereus* MH778713 favoured seedling growth and helped tomato plants to manage *Fusarium* wilt.

## MATERIALS AND METHODS

### PCR amplification and sequencing of *rpoB* genes

Total DNA of *Bacillus* sp. MH778713 was extracted using the Wizard Genomic DNA Purification Kit (Promega Corporation), according to the manufacturer´s protocol. PCR was performed in a 2400 GeneAmp PCR Systems® Perkin Elmer thermocycler to amplify the *rpoB* gene using the rpoB83F primer 5′‐CCTSATCGAGGTTCACAGAAGGC‐3′ and the rpoB1061R primer 5′‐AGCGTGTTGCGGATATAGGCG‐3′ (Martens et al., 2008). Internal fragments of the *rpoB* gene (RNA polymerase, beta subunit) were amplified using the following PCR cycling conditions: 5′ 96℃, × 35 (30″ 94℃, 30″ 59℃, 1′ 72℃), 3′ 72℃, ∞ 20℃. PCR products and their concentration were verified by electrophoresis on a 1% agarose gel stained with ethidium bromide. A molecular size marker (GeneRuler 1Kb DNA Ladder) was included to estimate the length of the amplification products. The amplified products were purified to remove salts, polymerase and excess of primers and nucleotides, using a QIAquick PCR purification kit (Qiagen) according to the manufacturer's instructions. The concentration of purified products was measured in a NanoDrop® ND‐1000 spectrophotometer (NanoDrop Technologies). Sequencing was performed by the Genoscreen company service (Applied Biosystems 3730XL DNA sequencer). Consensus sequences were obtained using the Auto‐Assembler software (Applied Biosystems).

### *rpo*B gene sequence alignment and phylogenetic analyses

The GenBank accession number for *Bacillus* sp. strain MH778713 RNA polymerase beta‐subunit gene (*rpo*B) is MW250916. *rpo*B gene sequences were compared with those of the selected strains, including type material available from the GenBank database at the time of writing. Nucleotide sequence alignments were made using CLUSTAL_X and corrected manually using GeneDoc (Thompson et al., 1997). Maximum parsimony (MP) analyses were performed with MEGA X ver. 10.1.8 (Kumar et al., 2018). MP tree is based on the *rpo*B gene sequences comparison. The relationship of *B*. *cereus* group members and related species was obtained using the subtree‐pruning‐regrafting (SPR) algorithm (Nei & Kumar, 2000). The most parsimonious tree with length = 4319 is shown in Figure S1. The consistency index was 0.270896 (0.267163), the retention index was 0.866596 (0.866596), and the composite index was 0.234757 (0.231523) for all sites and parsimony‐informative sites are in (parentheses). The percentage of replicate trees in which the associated taxa clustered together in the bootstrap test (1000 replicates) is shown next to the branches, and only values greater than 70% were shown (Kumar et al., 2018; Tamura et al., 2011).

### Alkaline serine protease, endo and exochitinase amino acid sequence alignment and phylogenetic analyses

Maximum parsimony trees were generated based on the amino acid sequences of endo and exochitinase proteins coded in the genomes of *Bacillus* species, available from the GenBank database (https://www.ncbi.nlm.nih.gov/) at the time of writing. The evolutionary history was inferred using the MP method. The MP trees were obtained using the SPR algorithm (Nei & Kumar, 2000) with search level 1 in which the initial trees were obtained by the random addition of sequences (10 replicates). Evolutionary analyses were conducted in mega X (Kumar et al., 2018). For alkaline serine protease, a Neighbour‐Joining analysis was performed. The percentage of replicate trees in which the associated taxa clustered together in the bootstrap test (2000 replicates) were shown next to the branches. Evolutionary analyses were conducted in mega X (Kumar et al., 2018).

### Preparation of bacterial and fungal cultures

*Bacillus cereus* MH778713, isolated from *P*. *laevigata* nodules (Ramírez et al., 2019), was streaked onto yeast‐extract mannitol agar (YMA) plates and incubated for 24 h at 30℃ before their use. *Bacillus* *cereus* MH778713 grown in YM liquid medium was used to prepare bacterial suspensions in sterile distilled water for the crop protection assays. The ingredients (g/L) of YMA are yeast extract 1.0, mannitol 10, dipotassium phosphate 0.5, sodium chloride 0.1, glutamic acid 0.01 and agar 14. The fungal pathogen, *F. oxysporum*, was isolated from wilted tomato plants. A pathogenicity test was previously conducted using the root‐dipping inoculation method on tomato seedlings (Arenas et al., 2018). The fungus was grown on potato dextrose agar at 28℃ for a week before its use in the anti‐fungal assays. PDA was prepared as follows: 200 g peeled potato, 20 g agar and 20 g dextrose adjusted with distilled water to a final volume of 1 L.

### *In vitro* bacterium–fungus interaction assays

#### Dual culture assay

The antagonistic activity of *Bacillus cereus* MH 778713 against *F*. *oxysporum* was assayed by dual culture technique in PDA adjusted to pH 6.0. In this method, biocontrol is due to diffusible compounds produced by the bacterial strain. Ten microlitres (μl) of a conidial suspension containing 1 × 10^6^ conidia per ml of *F*. *oxysporum* was seeded on one side of the plate and 10 μl of *B*. *cereus* MH778713 overnight liquid culture (1 × 10^10^ CFU per ml) was seeded on the other half. The plates were incubated for five days at 28℃. The experiment was done by triplicates, each treatment had five replicates. A PDA Petri‐plate inoculated with the fungus alone was used as a control to compare the mycelial growth in the presence and absence of *B*. *cereus* MH778713.

#### Antagonism due to volatile compounds emitted by *B. cereus* MH778713

To evaluate the antifungal activity of volatiles released by *B*. *cereus* MH778713 centrally partitioned Petri dishes were used to co‐cultivate bacterial and fungal strains. Thus, physical contact between them was prevented. PDA (10 ml) was poured into one side of the partitioned Petri dish for the growth of fungus and the other side of the plate was filled with 10 ml YMA for bacterial growth. Similar to dual culture assay, 10 μl of conidial suspension (1 × 10^6^ conidia per ml) was seeded on one side of the partitioned plate and 10 μl of *B*. *cereus* MH778713 overnight liquid culture (1 × 10^10^ CFU per ml) was seeded on the other half. The plates were sealed with parafilm and incubated at 28℃ in dark for 3 days. Partitioned Petri‐plates inoculated with the fungus alone were used as a control to compare the mycelial growth in the presence and absence of *B*. *cereus* MH778713 volatiles. Following 3 days of incubation, the inhibitory effect of bacterial volatiles on *F*. *oxysporum* was recorded in terms of radial growth and reduction in dry fungal biomass as compared to the control. For fungal dry biomass observations, the PDA compartment of the Petri plate was emptied onto a preweighed paper filter and dried at 60℃ for 24 h. Each experiment was performed with five replicates for each treatment.

### Biological control of *Fusarium* wilt on tomato in greenhouse conditions

Tomato seeds (*Lycopersicon esculentum* variety Marmande VR) susceptible to *F*. *oxysporum* f. sp. *lycopersici* (FOL) races 2 and 3 were used (McGrath et al., 1987). Seeds were surface‐sterilized by soaking them in 6% sodium hypochlorite for 15 min, then washed five times with sterile distilled water. Tomato seeds were germinated in a seedbed with sterile vermiculite‐peat moss (1:2 v/v) substrate. Twenty‐one days after sowing, seedlings at the third‐leaf stage were transferred to pots of 6.5 cm diameter containing 84 g of the sterile substrate (vermiculite‐peat moss 1:2 v/v). Pots containing five plants were kept under greenhouse conditions for 15 days with an ambient temperature between 14 and 25℃. The randomized complete block design was as follows. Treatment 1: tomato plants were inoculated with *B*. *cereus* MH778713 on one side of each plant stem at the soil line (1 ml of *B*. *cereus* MH778713 suspension per pot containing 1 × 10^10^ CFU per ml), and 24 h after, plants were challenged with *F*. *oxysporum* (1 ml per pot of a conidial suspension containing 1 × 10^6^ conidia per ml), inoculated in the same way as *B*. *cereus* MH778713. Treatment 2: tomato plants were inoculated with *F*. *oxysporum* and *B*. *cereus* MH778713 at the same time. Treatment 3: tomato plants were first challenged with *F*. *oxysporum*, and 24 h after, plants were inoculated with B. *cereus* MH778713 suspension. Three types of control plants were included, the disease control which was inoculated with *F*. *oxysporum* alone. The bioagent control inoculated with *B*. *cereus* MH778713 alone and plant controls supplied with sterile distilled water. All plants were watered with sterile deionized water without any fertilizer. The experiment was repeated once with five replicates per treatment. Each replicate consisted of five plants per pot. The wilted plant percentage was evaluated a week after the fungal challenge.

A second crop‐protection experiment was performed with tomato plants of a bigger size. Tomato seeds were surface‐sterilized and sown in pots of 8 cm diameter containing 390 g of the sterile substrate (vermiculite‐peat moss 1:2 v/v). One plant per pot was grown under greenhouse conditions for 60 days with an ambient temperature between 12 and 24℃. At this time, three plants were inoculated with 1 ml of *B*. *cereus* MH778713 suspension per pot containing 1 × 10^10^ CFU per ml; three more plants were untreated and used as a negative control. After 24 h, all tomato plants were challenged with *F*. *oxysporum* (1 ml per pot of a conidial suspension containing 1 × 10^6^ conidia per ml) and kept for 35 more days. At this time, disease severity was calculated as the ratio of the number of leaves showing symptoms (wilting and yellowing of leaves) and the total number of leaves of each plant. All plants were watered with sterile deionized water without fertilization.

### Stimulation of plant growth by *B*. *cereus* MH778713 and its volatiles *in vitro* and *in vivo* assays

Tomato seeds (*L. esculentum* variety Marmande VR) were surface‐sterilized with sodium hypochlorite as described previously. For *in vitro* growth stimulation assays, seeds were germinated on partitioned Petri plates containing agar (0.75% w/v) on one side for seedling growth and YMA on the other side for *B*. *cereus* MH778713 growth. Partitioned Petri dishes containing seeds were incubated at 28℃ in darkness for 3 days for seed germination; after that, the other side of the Petri dish containing YMA was seeded, with 10 μl of *B*. *cereus* MH778713 (1 × 10^10^ CFU per ml) and incubated for 6 more days. Control seedlings were kept without *Bacillus* on the YMA side during the whole experiment. The stimulation of plant growth was recorded in terms of plant weight as compared to the controls. Each seedling treatment was done with five replicates.

For *in vivo* growth stimulation assays, sterile seeds were sown in pots of 6.5 cm diameter, containing 84 g of a sterile substrate (vermiculite‐peat moss 1:2 v/v), one seed per pot. Plants were grown under greenhouse conditions with an ambient temperature between 11 and 20℃ (this experiment was performed in wintertime). Tomato plants at the first set of true leaves were inoculated with *Bacillus* or exposed to *B*. *cereus* MH778713 volatiles, and control plants were kept untreated. After each treatment, plants were maintained in the greenhouse for 21 days, watering with sterile distilled water without fertilization, to determine plant growth promotion. Plant growth was evaluated by measuring the differences in dry and fresh green weights and by measuring the root and shoot lengths of the tomato seedlings. A randomized complete block design was performed. Treatments are described as follows: five plants were inoculated with 1 ml of *B*. *cereus* MH778713 suspension (1 × 10^10^ CFU per ml) on plant stem at the soil line, then the pots were fixed on the glass jars. For volatile plant stimulation, a YMA Petri plate containing *B*. *cereus* MH778713 was placed at the bottom of a glass jar and the pot with the plant was fixed on the top of the glass jar, sealed with parafilm to avoid the leaking of volatiles produced by *B*. *cereus*. Six holes (2 mm) were made in the bottom of the pots to allow the roots to be exposed to the bacterial volatiles or to drain the excess water. Every seven days, the Petri plate with *Bacillus* was replaced at the bottom of the jars. The pots containing the control plants (untreated) were also fixed on glass jars.

### The antifungal and plant‐growth‐promoting activity of hentriacontane, heneicosane, 2,4‐di‐tert‐butylphenol and tetracosane

Antifungal activity of hentriacontane, heneicosane, 2,4‐di‐tert‐butylphenol and tetracosane; was evaluated on PDA plates. Ten microlitres of *F*. *oxysporum* conidial suspension (1 × 10^6^ conidia per ml) was streaked in PDA Petri plates. After that, 5 mm diameter sterile paper discs with 20, 200 and 400 µg of each compound were placed on the plate to evaluate the antifungal activity. Petri plates were incubated at 28℃ for 3 days. The appearance of a zone of inhibition was recorded. Each experiment was repeated three times, with five replicates for each treatment.

Plant growth promotion was assayed on partitioned Petri plates containing agar (0.75% w/v) on one side for seedling growth and YMA on the other side. Partitioned Petri dishes containing seeds were incubated at 28℃ in darkness for 3 days for seed germination; after that, the other side of the petri dish containing YMA was inoculated with 50 μg of hentriacontane, heneicosane, 2,4‐di‐tert‐butylphenol or tetracosane and then incubated for 8 more days. Control seedlings were inoculated with 5 µl of water or benzene on the YMA side. Benzene was the solvent for each compound assayed. The stimulation of plant growth was recorded in terms of plant weight as compared to the controls. Each seedling treatment was done with five replicates.

### Skimmed milk agar plates

Skimmed milk agar was made as follows: 75 g of fat‐free milk powder (Svelty total move, Nestle) was reconstituted with 500 ml of distilled water. The mixture was autoclaved at 121℃ for 15 min. Similarly, 500 ml of 32% agar solution was autoclaved. After autoclaving and cooling to 60℃, both solutions were mixed to give a final concentration of 15 g/L agar and 75 g/L milk powder. After picking a colony (*B*. *cereus* MH778713) with a sterile toothpick, a milk agar plate was inoculated in the centre and incubated at 28℃ for 2 days. There were four replicates.

### Statistical analysis

The data obtained from fungal radial growth, plant growth promotion and percentage of wilted plants experiments were subjected to analysis using one‐way analysis of variance (ANOVA), and the significant differences between mean values were determined by the Tukey–Kramer Post Hoc tests using Microsoft 365 Excel for Windows. A significance level of *p* < 0.05 was used to determine the significant differences of data.

## RESULTS

### Molecular identification of *Bacillus cereus* MH778713

*Bacillus* sp. MH778713 was isolated from *P*. *laevigata* root nodules and previously identified belonging to the genus *Bacillus* clustering within the *Bacillus cereus* group (Ramírez et al., 2019). Because the evaluation of 16S rDNA sequences lacks specificity at the species level, the *rpoB* housekeeping gene was used, in the identification of *Bacillus* sp. MH778713 strain. The MP phylogenetic analysis (Figure S1) suggested that the *Bacillus* sp. MH778713 strain belongs to the species *B*. *cereus*.

### *In vitro* antagonistic response of *B. cereus* MH778713 towards *F. oxysporum*

**FIGURE 1:**
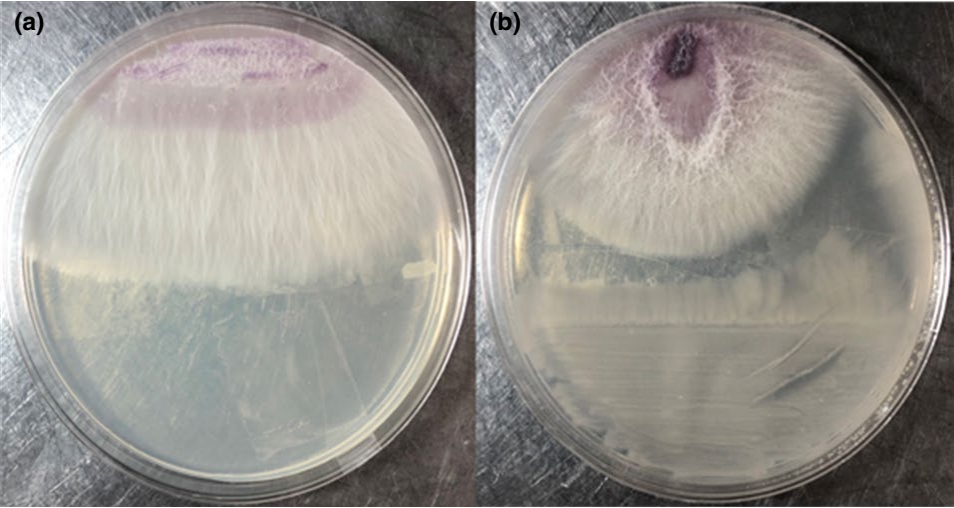
Antagonistic interaction between *Bacillus cereus* MH778713 and *Fusarium* strains over 5 days of growth. (a) *Fusarium oxysporum* alone as a control, showing normal growth. (b) Dual culture assay of *B*. *cereus* MH778713 and *F*. *oxysporum*. The experiment was done by triplicates, each treatment with five replicates

Rhizobacteria can elicit ISR to biotic and abiotic stresses. *Bacillus* *cereus* MH778713 has already shown the ability to break seed dormancy under chromium stress (Ramírez et al., 2020). In this work, the antagonistic traits of *B*. *cereus* MH778713 against a phytopathogenic *F*. *oxysporum* strain was evaluated (Figure 1). Antagonism assay in PDA plates revealed the antimicrobial activity of *B*. *cereus* MH778713 against *F*. *oxysporum*, a pathogen of tomato vascular wilt reported by Arenas et al. (2018).

To determine whether volatiles emitted by *B*. *cereus* MH778713 can negatively affect mycelial radial growth of *F*. *oxysporum*, co‐cultivations of *B*. *cereus* MH778713 with *F*. *oxysporum* were carried out in partitioned Petri dishes over three days (Figure S2). Control without *Bacillus* was also included (Figure S2A). The *F*. *oxysporum* radial growth with and without *B*. *cereus* MH778713 volatiles was different (*p* ≤ 0.05) with 1.53 ± 0.06 and 2.47 ± 0.05 cm. The dry biomass of mycelium with and without bacterial volatiles was recorded as 0.110 ± 0.004 and 0.153 ± 0.008 g. The results showed that *B*. *cereus* MH778713 is a *Fusarium* antagonistic bacterium whose volatiles affected the mycelial growth of *F*. *oxysporum*.

### Protective effect of *B. cereus* MH778713 against *F. oxysporum* wilt of tomato under greenhouse conditions

**FIGURE 2:**
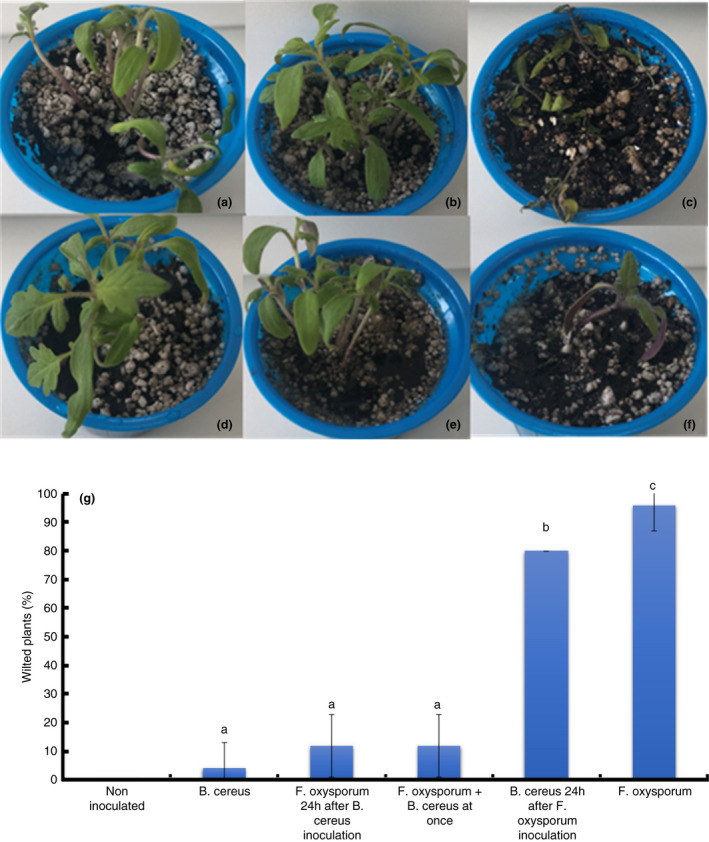
*Fusarium oxysporum* wilt of tomato under greenhouse conditions. The treatments were uninoculated control (a), inoculation with *Bacillus cereus* MH778713 (b), inoculation with *F*. *oxysporum* (c), inoculation of *B*. *cereus* MH778713 first and *F*. *oxysporum* 24 h later (d), co‐inoculation of *B*. *cereus* MH778713 and *F*. *oxysporum* at once (e) and inoculation of *F*. *oxysporum* first and *B*. *cereus* MH778713 24 h later (f). (g) Graphical representation of the percentage of wilted plants in response to different treatments (a–f). One‐way ANOVA analyses were performed, and a Tukey–Kramer post hoc test was used to identify inoculation treatments with means significantly different from the uninoculated control at *p* ≤ 0.05

*Bacillus* *cereus* MH778713 was assayed for its capability to manage the tomato *Fusarium* wilt. For that purpose, tomato plants were co‐inoculated with the pathogen (*F*. *oxysporum*) and biocontrol agent (*B*. *cereus* MH778713) in different combinations. In the first treatment, tomato plants were infected, with *F*. *oxysporum* 24 h after *B*. *cereus* MH778713 inoculation (Figure 2d). In the second treatment, tomato plants were inoculated with *F*. *oxysporum* and *B*. *cereus* MH778713 at the same time (Figure 2e). In the third treatment, tomato plants were inoculated with *B*. *cereus* MH778713 24 h *after* the *F*. *oxysporum* infection (Figure 2f). Compare to the disease control (Figure 2c), which was inoculated with a pathogenic strain of *F*. *oxysporum*, all combinations of *B*. *cereus* MH778713 and *F*. *oxysporum* reduced the number of wilted plants (Figure 2g), suggesting the protective effect of *B*. *cereus* MH778713. There was no difference in wilting between the control supplied with water (Figure 2a) and the bioagent control inoculated with *B*. *cereus* MH778713 (Figure 2b); indicating that the MH778713 strain was not phytopathogenic.

**FIGURE 3:**
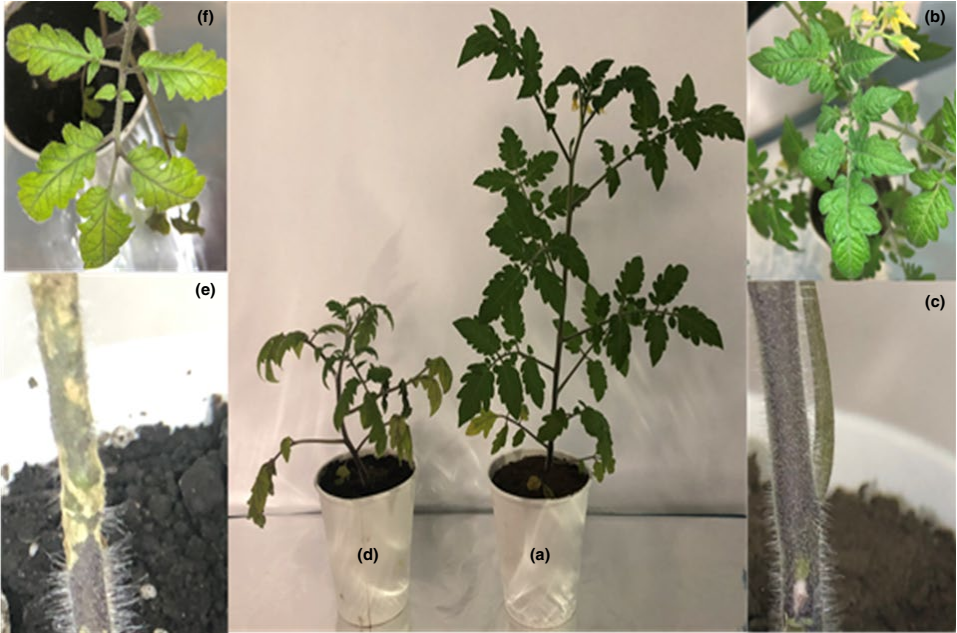
*Bacillus cereus* MH778713‐treated plants showing less disease severity compared to plants inoculated with *Fusarium oxysporum* alone. (a) Bacterial‐treated plant challenged with *F*. *oxysporum*. (b) Top‐view of *B*. *cereus* MH778713‐treated plant confronted with *F*. *oxysporum*. (c) Stem of the bacterial‐treated plant challenged with *F*. *oxysporum*. (d) Tomato plant infected with *F*. *oxysporum*. (e) Stem of tomato plant infected with *F*. *oxysporum*. (f) Top‐view of tomato plant infected with *F*. *oxysporum*. Three plants of the same size were used for each treatment

Crop‐protection was also assayed in older tomato plants (Figure 3). Six plants 60 days after sowing were used, three of them were inoculated with *B*. *cereus* MH778713 and three were not. After 24 h of *B*. *cereus* MH778713 inoculation, all plants were challenged with *F*. *oxysporum*. After fungal inoculation, plants were kept in the greenhouse for 35 more days. *Fusarium* *oxysporum* infected plants treated with *B*. *cereus* MH778713 (Figure 3a) were larger and greener than control plants infected with *F*. *oxysporum* (Figure 3d). Stem damage was observed in control plants infected with *F*. *oxysporum* (Figure 3e), but not in *F*. *oxysporum* infected plants treated with *B*. *cereus* MH778713 (Figure 3c). The disease severity was analysed. Disease severity of 88.1 ± 4.1% was observed in control plants while treated plants with bacterial strain presented only 23 ± 8.2% of disease severity.

### Plant‐growth‐promoting activity of *B. cereus* MH778713 *in vitro* and *in vivo* tests

**FIGURE 4:**
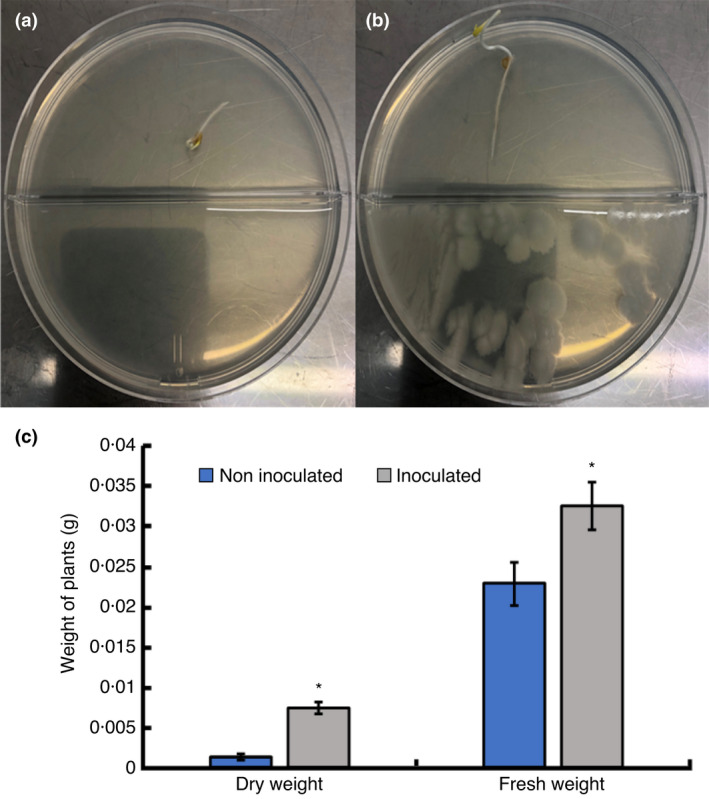
Effects of volatiles produced by *Bacillus cereus* MH778713 on the growth promotion of tomato seedlings in partitioned Petri dish. (a) Untreated control seedlings. (b) Seedlings exposed to *B*. *cereus* MH778713 volatiles over 6 days. (c) The fresh and dry weight of plants. The asterisks indicate that volatiles produced by *B*. *cereus* MH778713 had a positive effect on the growth of tomato seedlings. Each seedling treatment was done with five replicates

To test whether volatiles produced by *B*. *cereus* MH778713 can promote tomato seedling growth, co‐cultivations of *B*. *cereus* MH778713 and tomato seedlings in partitioned Petri dish were carried out over 6 days (Figure 4). As shown in Figure 4b, the size of seedlings exposed to the volatiles released from *B*. *cereus* MH778713 was larger than those of untreated seedlings (Figure 4a), reflecting on the weight of plants (Figure 4c). Results demonstrated the plant growth promotion elicited by *B*. *cereus* MH778713 volatiles.

**FIGURE 5:**
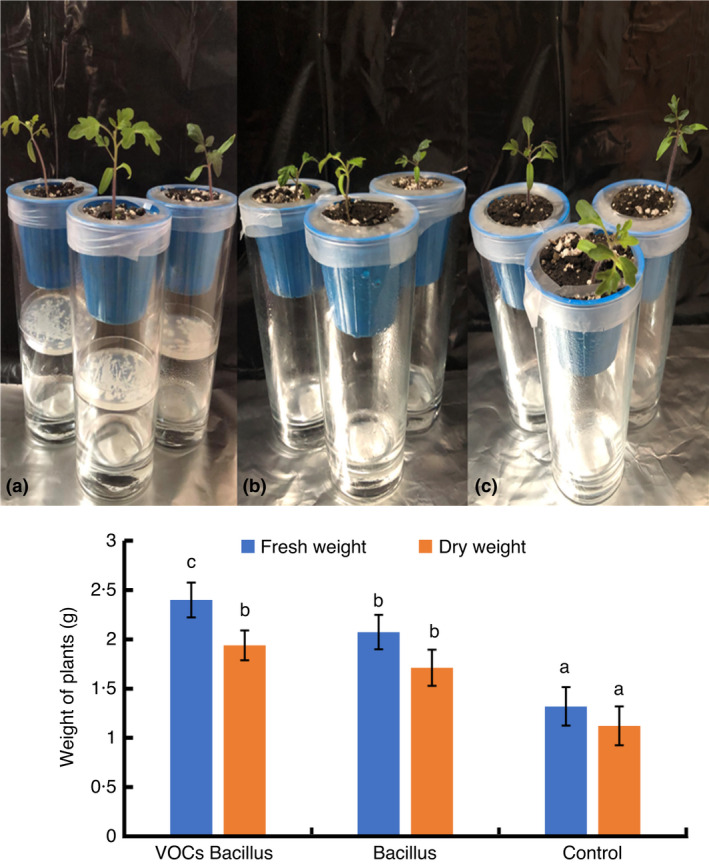
*Bacillus cereus* MH778713 plant growth promotion under greenhouse conditions. (a) Tomato plants were exposed to the volatile organic compounds produced by *B*. *cereus* MH778713. Petri plates inoculated with *Bacillus* were placed at the bottom of the glass jars, allowing volatiles to stimulate the roots through the small (2 mm) holes in the bottom of the pots. (b) Untreated tomato plants were used as controls. (c) Tomato plants inoculated with *B*. *cereus* MH778713. Five plants at the first set of true leaves were inoculated or exposed to *B*. *cereus* volatiles, the pots fixed in the jars were incubated in the greenhouse for 21 days. Dry and fresh weight was evaluated. The different letter means a significant difference at *p* ≤ 0.05

To determine whether *B*. *cereus* MH778713 can enhance plant growth *in vivo*, five tomato plants were inoculated with *B*. *cereus* MH778713 or exposed to its volatiles. Treated tomato plants were grown under greenhouse conditions for 21 days and were compared to the untreated control (Figure 5). The shoot length of plants treated with volatiles or inoculated with *B*. *cereus* MH778713 was 7.8 ± 0.12 cm and 7.38 ± 0.08 cm, while the shoot length of control plants was 2.14 ± 0.40 cm. The small size of these plants can be attributed to the low temperature of the wintertime of this experiment. The root length of plants exposed to volatiles (7.56 ± 0.15 cm) or inoculated with *B*. *cereus* MH778713 (7.24 ± 0.09 cm) was also stimulated, as compared to the controls (1.12 ± 0.08 cm). The difference in size was reflected in the fresh and dry weight of plants, with statistical significance (Figure 5). The *in*‐*planta* experiments showed that *B*. *cereus* MH778713 promotes plant growth by the emission of volatiles and by the direct inoculation in the root system.

### Antifungal and plant‐growth‐promoting activity of heneicosane, 2,4‐di‐tert‐butylphenol, hentriacontane and tetracosane

*Bacillus* *cereus* MH778713 releases the 2,4‐di‐tert‐butylphenol, heneicosane, hentriacontane and tetracosane. Those compounds break the dormancy of *P*. *laevigata* and *A*. *thaliana* seeds subjected to chromium‐stress (Ramírez et al., 2020). To evaluate whether those compounds, could also inhibit the growth of *F*. *oxysporum*, dual cultures in PDA plates were performed using paper discs with 20, 200 and 400 µg of each compound (Figure S3). Only hentriacontane and 2,4‐di‐tert‐butylphenol at 400 µg inhibited the growth of *F*. *oxysporum*, as denoted by the zone of inhibition (Figure S3a,b).

**FIGURE 6:**
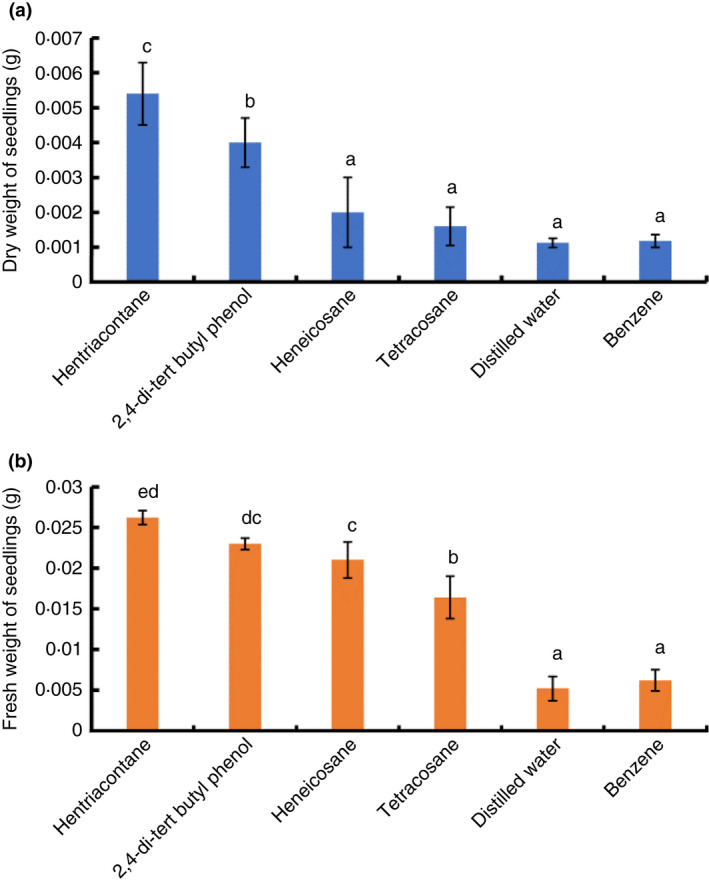
Growth stimulation of tomato seedlings by volatiles of 2,4‐di‐tert‐butylphenol, heneicosane, hentriacontane and tetracosane in partitioned Petri dishes. (a) The dry weight of seedlings exposed to volatiles for 8 days. (b) Fresh weight of seedlings exposed to volatiles for 8 days. Water and benzene were included as controls. Each seedling treatment was done with five replicates. The different letters mean a significant difference at *p* ≤ 0.05

It was reported that 2,4‐di‐tert‐butylphenol, heneicosane, hentriacontane and tetracosane break seed dormancy through airborne signals; therefore, their volatiles were tested for plant‐growth promotion (Figure 6). Tomato seedlings were exposed to the volatiles of 50 µg of 2,4‐di‐tert‐butylphenol, heneicosane, hentriacontane and tetracosane in partitioned Petri dishes over 8 days. The dry and fresh weight of seedlings expose to 2,4‐di‐tert‐butylphenol and hentriacontane were larger than those of control seedlings (Figure 6). Since all compounds were dissolved in benzene, it was tested as a control besides distilled water (Figure 6). Results demonstrated that volatiles of 2,4‐di‐tert‐butylphenol and hentriacontane enhanced plant growth.

### Alkaline serine protease, endo and exochitinase phylogenetic analyses

It is known that *Bacillus* species have hydrolytic enzymes (chitinases, glucanases and proteases) to lyse fungal cell walls. A phylogenetic analysis of alkaline serine protease, endo and exochitinases was performed to estimate their diversity within *B*. *cereus* group. Forty‐eight endochitinase sequences coded in the genome of *Bacillus* species, with an approximate length of 675 amino acids, were obtained from the GenBank database. The phylogenetic analysis resulted in one most parsimonious tree (Figure S4). The endochitinases from the *Bacillus cereus* group were placed in one cluster and had 97% of bootstrap support. Thirty‐eight exochitinase sequences coded in the genome of *Bacillus* species, with an approximate length of 360 amino acids, were also obtained from the GenBank database. The phylogenetic analysis resulted in one most parsimonious tree (Figure S5). The exochitinases from the *Bacillus cereus* group were arranged in one cluster, with 99% of bootstrap support. Amino acid sequence analysis of both endo and exochitinase in the *Bacillus cereus* group; showed a high degree of conservation inside this group, at the amino acid level. Furthermore, an alkaline serine protease of *B*. *cereus* was found in the same cluster of the phylogenetic tree (Figure S6) with different strains, such as *B*. *cereus* OPD49186.1, *B*. *cereus* ASI72743.1, *B*. *cereus* ASK14498.1 and *B*. *thuringiensis* ARP57672.1. Finally, a skimmed milk agar assay suggested the potential protease activity of *B*. *cereus* MH778713 (Figure S7).

## DISCUSSION

The sequence alignment of the *rpoB* gene and MP phylogenetic analyses, performed in this work, confirmed the relationship of MH778713 strain with *B*. *cereus* species (Figure S1). Ramirez et al. (2019) reported the relationship of *Bacillus* sp. MH778713 strain with the *B*. *cereus* group through a maximum‐likelihood phylogenetic tree based on the 16S rRNA gene sequence. *Bacillus* *cereus* is well‐known for causing foodborne illness (Nguyen & Tallent, 2019). However, for agriculture, *B*. *cereus* strains hold potential applications since they are commonly found in soil, being part of the root‐associated microbiomes. Several *B*. *cereus* strains promote plant growth and elicit ISR against plant pathogens (Chang et al., 2007; Niu et al., 2011; Romeiro et al., 2010; Wang et al., 2019). In line with these reports, *B*. *cereus* MH778713 displayed a protective effect against tomato *Fusarium* wilt (Figures 2 and 3). Moreover, *B*. *cereus* MH778713 promoted tomato plant growth through the volatiles or by the direct inoculation in the root system (Figure 5). *Bacillus* *cereus* MH778713 enhances *P*. *laevigata* growth in Jensen's nitrogen‐free medium and assists plant‐growth and seed germination under chromium stress (Ramírez et al., 2019, 2020). The phytopathogenic protecting traits described here, the ability to promote plant‐growth with (Ramírez et al., 2019) and without Cr‐stress (Figure 5), and the beneficial interaction with legumes and nonlegumes, enable *B*. *cereus* MH778713 as an outstanding PGPR to boost crop growth and disease resistance.

Legumes‐associated rhizobacteria have shown a positive influence on plant growth of both legumes and nonlegumes (Subramanian et al., 2016). In this research, *B*. *cereus* MH778713, a root‐nodule isolated bacterium, showed the ability to protect tomato against *Fusarium* wilt (Figures 2 and 3). The timely administration of a biocontrol agent is of extreme importance to suppress disease development. Applying the biocontrol agent after pathogen infection significantly decreases biocontrol efficacy for a biocontrol agent with the competition or induced resistance as the primary mechanism of control (Xu et al., 2010). Thus, the protective effect of *B*. *cereus* MH778713 was less effective when applied after the fungal pathogen (Figure 2f), suggesting that *B*. *cereus* MH778713 might possess either competition or induced resistance as the main mechanism of biological control.

The crop protection against phytopathogenic fungus includes the production of cell wall‐degrading enzymes such as chitinases, proteases, cellulases or glucanases. The induction of systemic resistance, competition, the production of lipopeptide, phthalic acid compounds and bacteriocins (Chang et al., 2007; Ferraz et al., 2014; Niu et al., 2011; Romeiro et al., 2010). During dual culture assay (Figure 1), antifungal activity of *B*. *cereus* MH778713 could be attributed to the production of diffusible metabolites and/or hydrolysing enzymes (Miljaković et al., 2020). We found alkaline serine protease, exo and endochitinases in the genome of the *B*. *cereus* group (Figures [Link], [Link], [Link]), besides proteolytic activity on skimmed milk agar assay (Figure S7). The *B*. *cereus* group includes several *Bacillus* species with closely related phylogeny, such as *B*. *cereus*, *B*. *thuringiensis*, *B*. *anthracis*, *B*. *toyonensis*, *B*. *mycoides*, *B*. *pseudomycoides*, *B*. *weihenstephanensis* and *B*. *cytotoxicus* (Ehling‐Schulz et al., 2019). The strains of the *B*. *cereus* group have been reported for their chitinolytic and proteolytic activities. For example, the chitinase from *B*. *thuringiensis* subsp. colmeri prevented the germination of fungal spores (Liu et al., 2010). *Bacillus* *mycoides* strain BacJ elicits systemic resistance in sugar beet which was related to increased activity of peroxidase, β‐1,3‐glucanase and chitinase (Bargabus et al., 2002). Considering that bacterial chitinases are implicated in the breakdown of micelles of fungal pathogens (Hamid et al., 2013; Wang et al., 2002), that many *Bacillus* species produce fungal wall hydrolysing enzymes (Shafi et al., 2017) and that amino acid sequences of chitinases and serine protease in databases show that these proteins are coded in the genome of bacillus species closely related with *B*. *cereus*, we can propose that antifungal activity of *B*. *cereus* MH778713, against *F. oxysporum*, could be partially attributed to the production of hydrolytic enzymes. Furthermore, not only chitinases are produced by bacteria belonging to the *B*. *cereus* group, other enzymes such as chitosanases, β‐1,3 glucanases and alkaline proteases are also coded in the genomes of *Bacillus* species (data not shown), representing the great potential for the crop management of fungal pathogens.

The interest of our research group is the study of volatiles produced by *B*. *cereus* MH778713. Thus, co‐cultivations of *B*. *cereus* MH778713 and *F*. *oxysporum* were performed in partitioned Petri dishes to test volatiles fungistatic activity (Figure S2). *Bacillus* *cereus* MH778713 volatiles reduced the mycelial radial growth of *F*. *oxysporum* by 38%. Although *B*. *cereus* and other *Bacillus* species produce antifungal volatiles (Chaves‐López et al., 2015; Gao et al., 2018; Khan et al., 2018), their diffusible metabolites are more antagonistic than these volatile compounds (Abdallah et al., 2016). Among the reported bacterial volatiles with antifungal activity are 2,4‐di‐tert‐butylphenol, benzothiazole, propanone, 3‐methyl‐1‐butanol, 2‐methyl propanoic acid and 3‐methyl butanoic acid. Volatiles released from *B*. *cereus* MH778713 scarcely inhibited the growth of *Fusarium* (Figure S2) and promoted a higher growth of tomato seedlings compared to the controls (Figures 4c and 5a). *Bacillus subtilis* strain GB03 and *B*. *amyloliquefaciens* strain IN937a promote the growth of *A. thaliana* by releasing 2,3‐butanediol and acetoin as volatiles, while *Bacillus megaterium* Strain XTBG34 do it by releasing 2‐pentylfuran (Lee et al., 2012; Ryu et al., 2003; Zou et al., 2010). 2‐nonanone and 2‐undecanone produced by *Bacillus* sp. BCT9 are growth inducers of horticultural species (Fincheira et al., 2017). In our study, hentriacontane and 2,4‐di‐tert‐butylphenol released from *B*. *cereus* MH778713 stimulated the growth of tomato seedlings (Figure 6).

Previous studies have reported that 2,4‐di‐tert‐butylphenol has antifungal activity (Gao et al., 2018). 2,4‐di‐tert‐butylphenol inhibits the growth and spore germination of *F*. *oxysporum* with similar efficiency as the commercial fungicide Bavistin (Dharni et al., 2014). In this study, the antifungal activity of 2,4‐di‐tert‐butylphenol was confirmed, besides its ability to stimulate plant growth. Surprisingly, hentriacontane antifungal activity was the highest of all compounds tested (Figure S3a). Hentriacontane has been identified in the crude extract of *Paracoccus pantotrophus* FMR19 and the volatiles emitted by *Pseudomonas brassicacearum* USB2104 (Giorgio et al., 2015; Faridha Begun et al., 2016); its antimicrobial activity has not been studied before but inferred from the antimicrobial activity of a cuticular wax constituted mainly of hentriacontane (Olubunmi et al., 2009). Further studies should be performed to elucidate the mechanism of the antifungal activity of the hentriacontane.

The mechanism of action of inhibition of *F*. *oxysporum* by *B*. *cereus* MH778713 remains to be fully elucidated. Although volatile components may have some role, other antifungal metabolites such as lipopeptides, toxins and hydrolytic enzymes could play a significant part. Additional field experiments are needed to determine the effectiveness of *B*. *cereus MH778713* as a biological control agent against *Fusarium* wilt of tomato.

In summary, *B*. *cereus* MH778713 and its volatiles stimulated tomato seedling growth and protected tomato plants against *Fusarium* wilt in greenhouse experiments. The beneficial interaction between *B*. *cereus* MH778713 and tomato seedlings demonstrated the potential of *B*. *cereus* MH778713 as a biocontrol agent.

## CONFLICT OF INTEREST

No conflict of interest declared.

## AUTHOR CONTRIBUTIONS

VR, JAM and AB conceived, designed and directed the project, contributed to the interpretation of the results and designed the figures. VR, JM, DCA and MRBC participated in the acquisition of the data and developed some methodologies. AB took the lead in the writing of the manuscript. All authors provided critical feedback, helped to shape the research, discussed the results and contributed to the final version of the manuscript.

## ETHICAL STATEMENT

No animal uses.

## Supporting information

Figure S1

Figure S2

Figure S3

Figure S4

Figure S5

Figure S6

Figure S7
