# Supplementary figures and images for: Bacillus cereus MH778713 elicits tomato plant protection against Fusarium oxysporum
Source: J Appl Microbiol. 2021 Jul 6;132(1):470–82. doi: 10.1111/jam.15179 (PMC9291537; doi:10.1111/jam.15179)

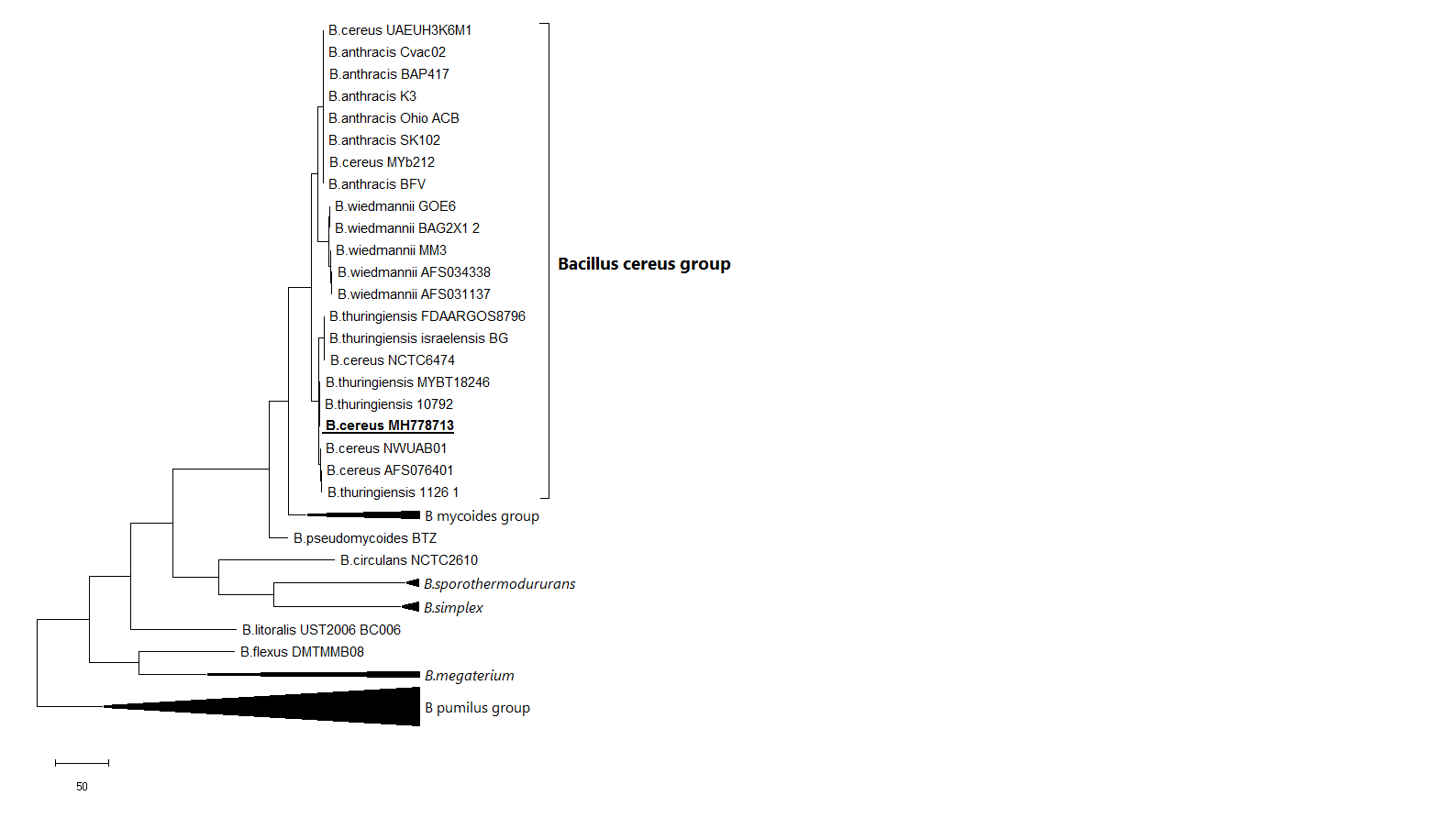

Supplement: Supplementary file 1 — Figure S1 [file JAM-132-470-s005.tif]

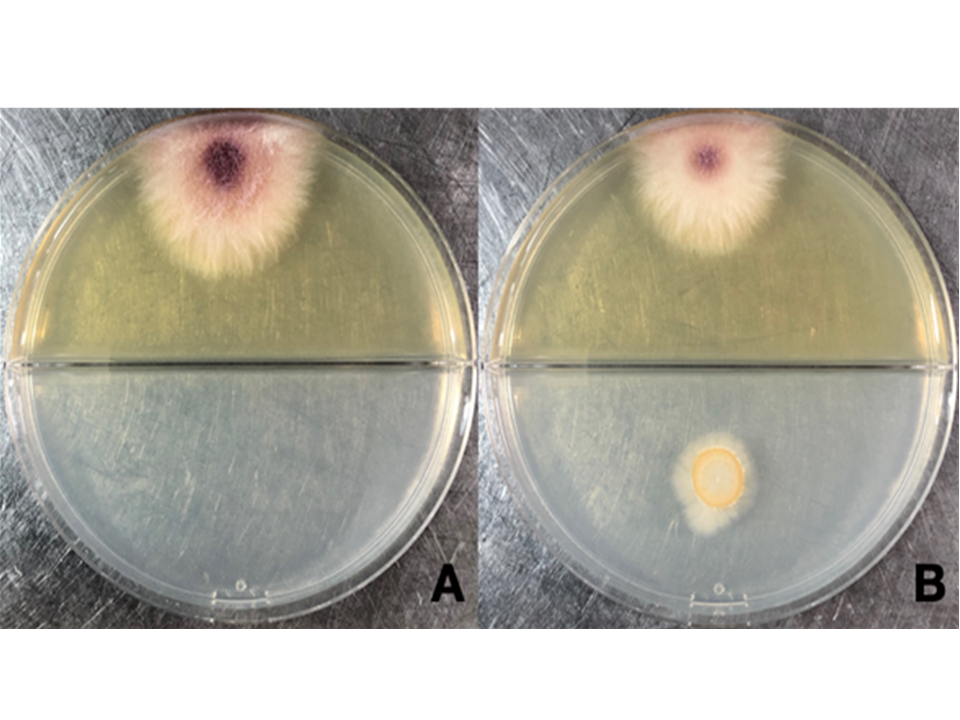

Supplement: Supplementary file 2 — Figure S2 [file JAM-132-470-s001.png]

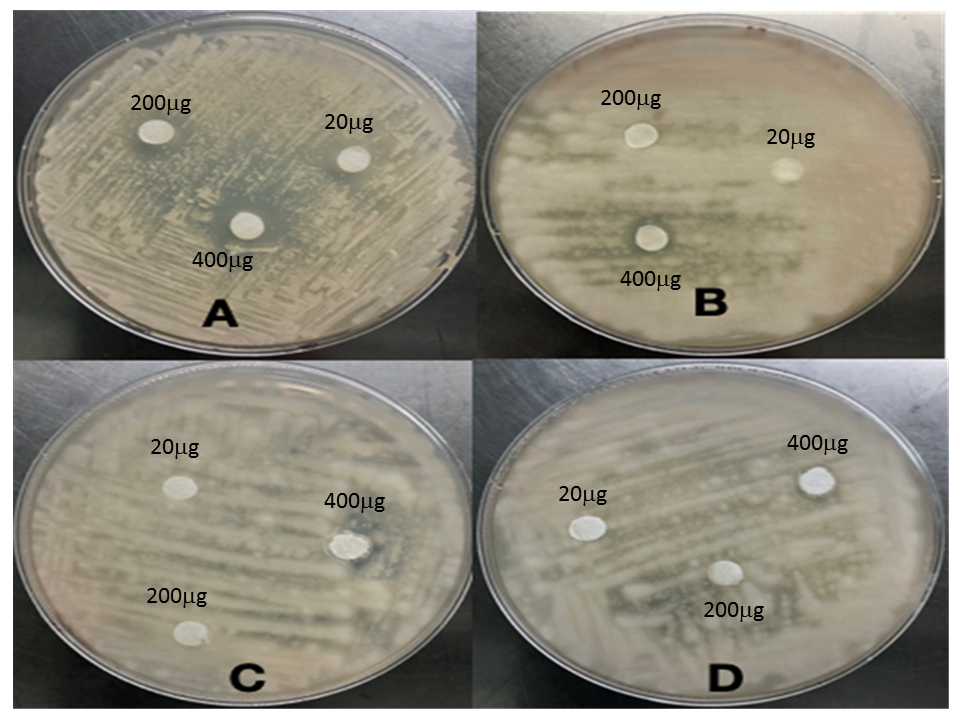

Supplement: Supplementary file 3 — Figure S3 [file JAM-132-470-s004.png]

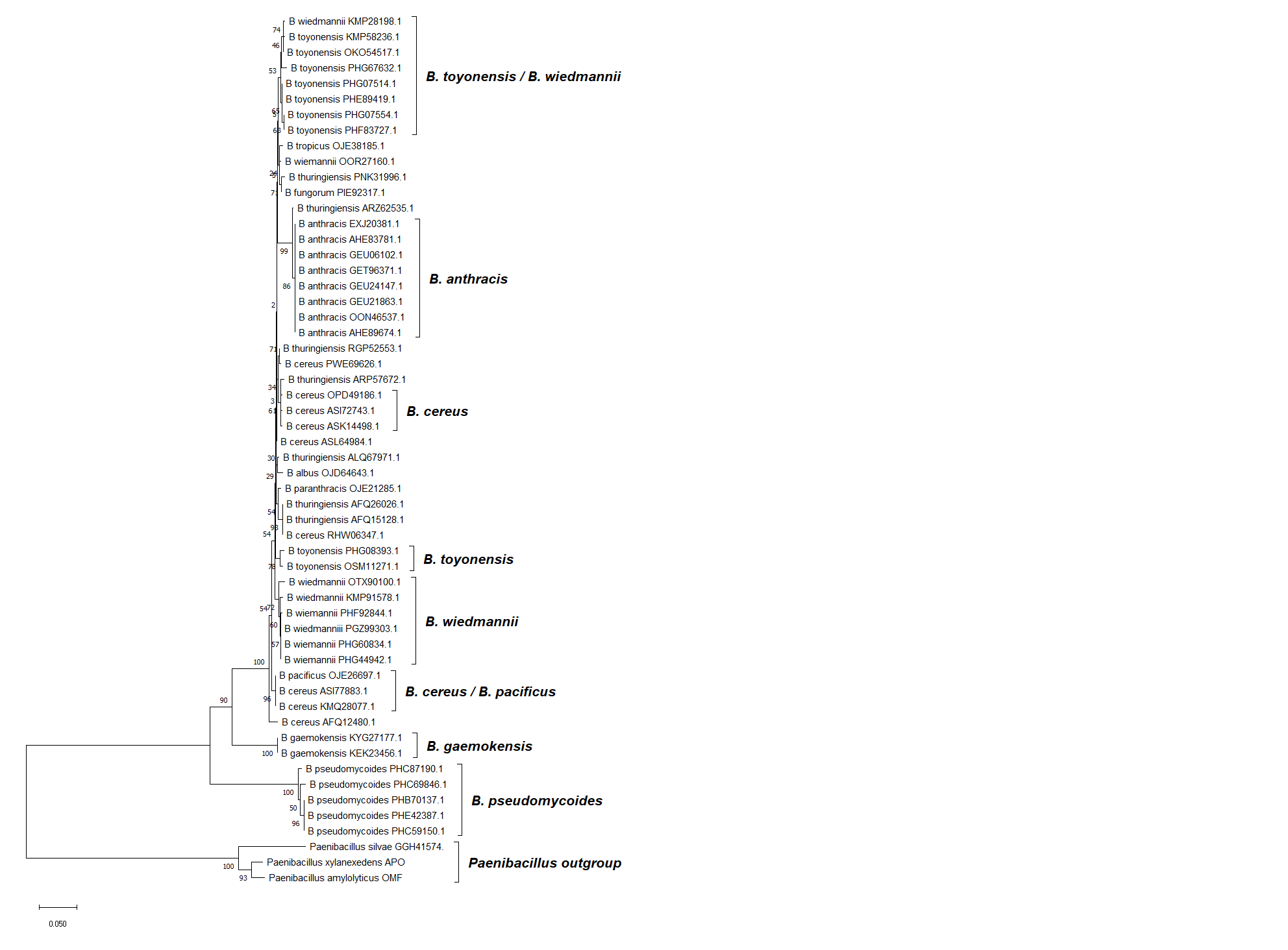
Fig S6. Neighbor-Joining analysis of **alkaline serine** **protease** in *Bacillus cereus* group.

Supplement: Supplementary file 6 — Figure S6 [file JAM-132-470-s002.docx]

## Slide 1
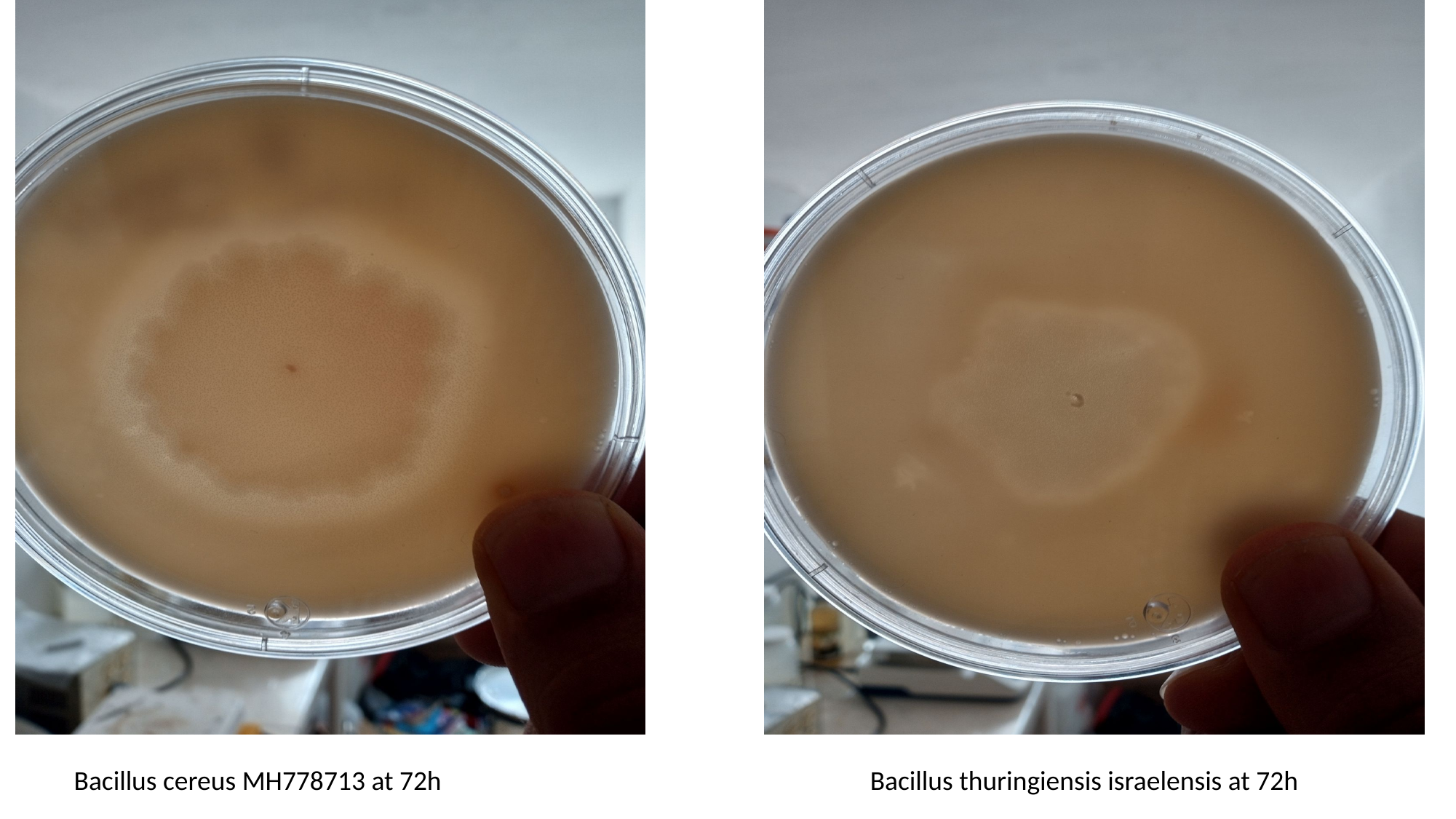

Bacillus cereus MH778713 at 72h
Bacillus thuringiensis israelensis at 72h

Supplement: Supplementary file 7 — Figure S7 [file JAM-132-470-s006.pptx]
