# Supplementary material for: Bacillus cereus MH778713 elicits tomato plant protection against Fusarium oxysporum
Source: J Appl Microbiol. 2021 Jul 6;132(1):470–82. doi: 10.1111/jam.15179 (PMC9291537; doi:10.1111/jam.15179)
Supplement: Supplementary file 4 — Figure S4 [file JAM-132-470-s007.docx]

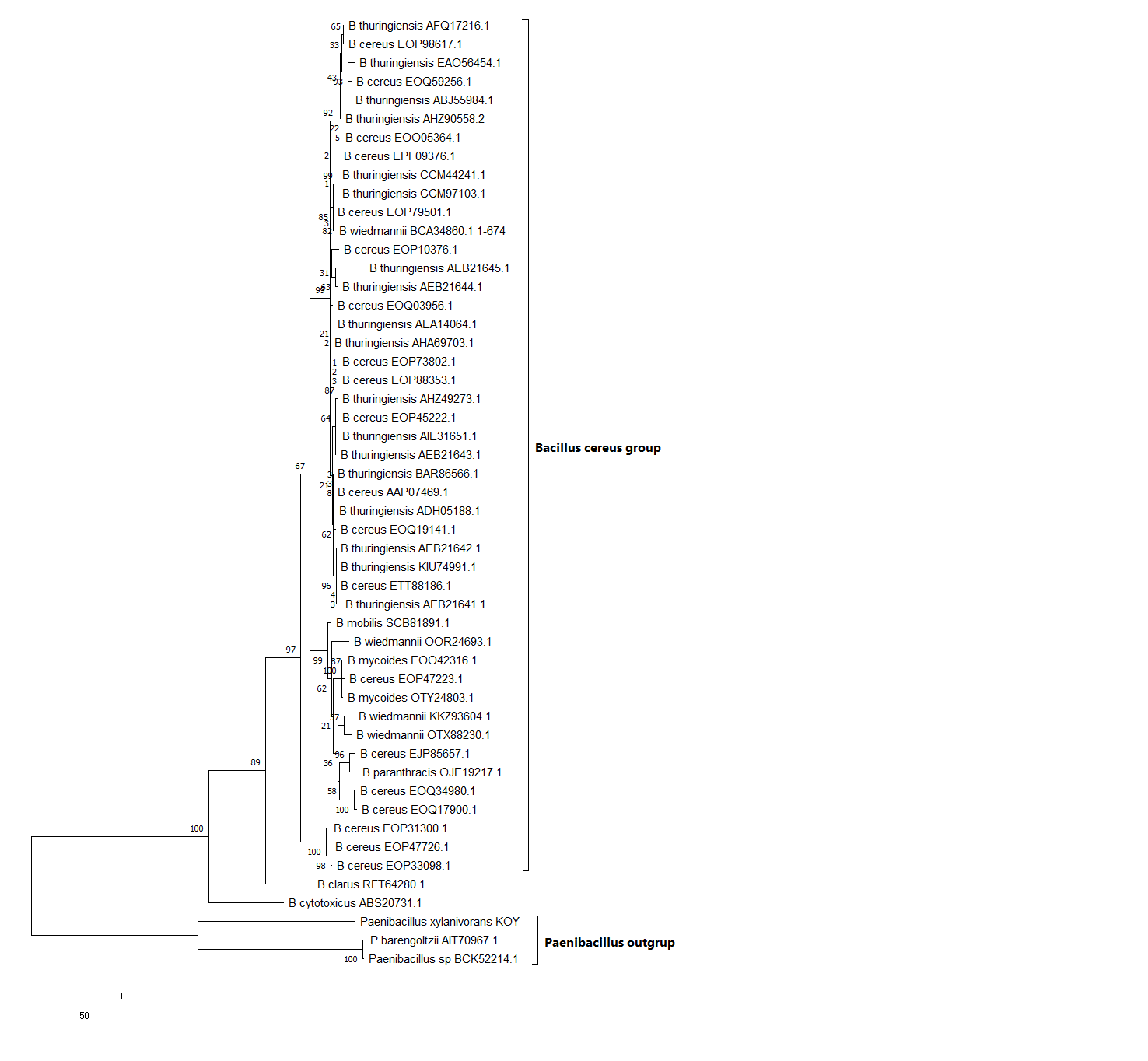


**Figure S4.** Maximum parsimony analysis of endochitinase proteins in *Bacillus cereus* group. The MP tree is based on the endochitinase amino acid sequences comparison, showing the relationship of endochitinase proteins coded in the genomes of *Bacillus* species.
